# Incidence of and social-demographic and obstetric factors associated with postpartum depression: differences among ethnic Han and Kazak women of Northwestern China

**DOI:** 10.7717/peerj.4335

**Published:** 2018-01-29

**Authors:** Ling Chen, Li Ding, Ming Qi, Chao Jiang, Xin-Min Mao, Wen-Zhi Cai

**Affiliations:** 1 Department of Nursing, Shenzhen hospital, Southern Medical University, Shenzhen, Guangdong, China; 2 Department of Cardiology, Xinjiang Medical University Affiliated Second Hospital, Urumqi, China; 3 Department of Rehabilitation and Psychology, First Affiliated Hospital of College of Medicine, Shihezi University, Shihezi, China; 4 Nursing Department, Fuyun People’s Hospital, Altay, China; 5 Reproductive Medicine Center, Xinjiang Medical University Affiliated First Hospital, Urumqi, China

**Keywords:** Postpartum depression, Health status disparities, Urinary incontinence, Risk factors, Race

## Abstract

**Background:**

Studies on postpartum depression (PPD) in China have focused primarily on women of Han ethnicity, whereas work on other ethnic groups has proven limited. This study explored the ethnic differences of associated social-demographic and obstetric factors for PPD between Han-majority and Kazak-minority women in northwestern China.

**Methods:**

Han and Kazak women who received routine examinations at four hospitals in a multi-ethnic area of China six weeks after childbirth between March 2016 and December 2016 were included in the study. Data on the women’s socio-demographic characteristics, obstetric factors, and possible depression at six weeks after childbirth were collected. We examined the associated factors of PPD using multivariable logistic regression analyses by ethnic group.

**Results:**

The overall incidence of PPD was 14.6% (184/1,263) at six weeks after childbirth. PPD was detected more frequently among Kazak (16.1%) than Han women (13.1%). Kazak women exhibited a higher risk of PPD (adjusted *OR* = 1.561, 95% CI [1.108–2.198], *P* = 0.011). Urinary incontinence (UI) represented a significant risk factor of PPD for Kazak compared with Han women (*OR* = 1.720, 95% CI [1.056–2.804], *P* = 0.003). In contrast, the presence of the mother-in-law as a caregiver after childbirth demonstrated a positive association with PPD among Han (*OR* = 2.600, 95% CI [1.499–4.512], *P* = 0.001), but not with Kazak women.

**Conclusions:**

Kazak women were more likely to develop PPD than Han women, even after controlling for confounders. Moreover, distinct risk factors for PPD existed for Han and Kazak women. Future research that explores the relationships between Han women and their mothers-in-law as well as Kazak women’s attitudes toward UI could help us further understand PPD in these populations.

## Introduction

Postpartum depression (PPD) is a common psychological problem that affects as many as 13–19% of women (O’Hara & McCabe, 2013). Generally, PPD occurs within the first four weeks after delivery (Gao et al., 2016), causing significant distress for the parents and adverse effects on the healthy development of their offspring. The pathogenesis of PPD is unknown, but psycho-social predictors, such as depression history, antenatal depression or anxiety, poor social support, marital dissatisfaction, and stressful life events, have been widely demonstrated by multiple meta-analyses (Beck, 2001; Robertson et al., 2004; O’Hara & Swain, 1996), while the contribution of social-demographic and obstetric factors differs by ethnicity and culture (Liu & Tronick, 2013; Wei et al., 2008).

Most research in China has focused on the largest ethnic group in China—the Han—whereas studies devoted to other ethnic groups are limited (Yan et al., 2015; Yang et al., 2015). Xinjiang, an area that is less developed in comparison to the Central and Eastern regions, is located in the northwest corner of China. The region is populated by a variety of ethnicities. The Kazaks, one of the largest minorities, has a population of 1.25 million (approximately 6.5% of the total population in Xinjiang province) according to the 2010 census; and the proportion for Kazaks is 6.5% (8.74 million) (Li et al., 2015). A considerable number of Kazaks are nomadic. Consequently, they have less access to medical resources. Postpartum mental health of Kazak women had not been documented.

This study was designed to observe and compare the incidence of and social-demographic and obstetric factors associated with PPD among Han and Kazak women. We hypothesized that social-demographic and obstetric factors are related to the development of PPD. The purpose of this study was to add information to the data on the risk profile of PPD by considering different ethnicities and cultures. In addition, we expected to alert scholars and medical practitioners to the health of minority females in Xinjiang, China, an economically underdeveloped region.

## Materials and Methods

### Ethics statement

Ethics approval for the study was granted by the Ethics Committee of the First Affiliated Hospital of Medicine College, Shihezi University (2015-134-01). All participants provided written informed consent.

### Estimation of sample size

Since the effect size of the difference between the PPD incidents of Han and Kazak ethnicity was unavailable in existing literature, the estimation of sample size was based on the standard practice of 1 variable per 10 events criterion for a binary logistic regression analysis (Rao, 2005). We took two steps to decide which risk factors to include in the present study. The first step was based on a comprehensive review of the literature (Fritel et al., 2016; Klainin & Arthur, 2009; Liu & Tronick, 2013; Templeton et al., 2003; Zaidi et al., 2017). Then, a focus group discussion with one obstetrician, two midwives, two practice nurses, and one psychiatrist was undertaken to explore their opinions based on the literature. We expected a total number of 16 possible candidate predictors for PPD based on the literature review conducted and consultation with clinical experts, which were age, race/ethnicity, religion, income, employment, parity, level of education, smoking, drinking, previous pregnancy loss, unplanned pregnancy, mode of delivery, infant gender, infant feeding, postpartum urinary incontinence (UI), and type of caregiver after childbirth. As no previous work in Kazak women had been undertaken, the estimate for the incidence of PPD was based on previous research among Chinese women, which is approximately 15% (Wan et al., 2009), the estimated sample size was 16 × 10/15% = 1,067. Considering the possibility of a negative response rate of 20%, the estimate of a final sample size was 1,334. To examine differences in incidence and associated factors of PPD between the Han and Kazak women, the study sample sought to represent an equal number of Han and Kazak participants.

### Participants

Purposive convenience sampling was employed to recruit participants. Women who visited obstetric clinics of four hospitals in Xinjiang province at six weeks postpartum were recruited consecutively between March 2016 and December 2016, with no compensation or honoraria. One of the four clinics was located in an area inhabited primarily by Hans, two of four clinics were located in an area inhabited by an approximately equal number of Han and Kazak women, and the remaining clinic was located in an area inhabited mainly by Kazaks. Access to medical care did not differ for the women with different lifestyles and cultural practices. Han and Kazak women were approached in the clinic waiting room by trained investigators. If interested, they were offered verbal and written information about the study. Women who provided written informed consent and were able to easily communicate with the investigators were asked to attend a face-to-face interview at the clinic immediately before or after their scheduled check. Women with a family history of mental illness or who had experienced mental disorders before or during pregnancy were excluded. A woman was deemed to have experienced a mental disorder if her answer was affirmative to one or both of the following questions: Have you ever seen a psychiatrist or psychologist before or during pregnancy? Have you ever taken antidepressants, anxiolytics or benzodiazepines before or during pregnancy? Women who provided incomplete/missing information, such refusal to give a response to one or more of the interviewer’s question, or did not remain throughout the duration of the investigation, were excluded.

### Data collection

An interview was conducted to obtain socio-demographic characteristics (ethnicity, age, religious faith, education, employment situation, household income per month, type of caregivers following childbirth, whether or not the pregnancy was planned), health-related behaviors (history of smoking and drinking), and obstetric factors (parity, previous pregnancy loss, mode of delivery on this occasion, infant gender, infant feeding, experience or not of UI after childbirth), and PPD. Most of the research was conducted by nurses whose mother tongue was Chinese. For the small number of Kazak women who could not understand and/or speak Chinese, assistance was provided by Kazak nurses.

### Definitions and descriptions of variables

The data collected on the different variables relied primarily on self-reported responses. Religious faith was defined as following or believing in the basic tenets of a major religion, such as Islam, Buddhism, or Christianity. Participants could choose between (1) religious faith and (2) no religious belief. Education level was separated into either (1) primary school or less or, (2) middle school or above. Participants were asked if they had steady employment or not. Steady employment was defined as full-time work eight months or more a year. Possible responses to the question about monthly household income was either 2,000 yuan (300 USD) or more or less than 2,000 yuan (300 USD). History of smoking was defined as more than one cigarette daily for more than six months and drinking was defined as equal or more than 50 ml daily or 500 ml weekly for more than 6 months. Previous pregnancy loss was defined as having a lost or terminated pregnancy, including miscarriage, induced abortion, or stillbirth. The existence of UI was self-reported involuntary urinary leakage after childbirth.

Postpartum depression was determined by the Chinese translation of the Edinburgh Postnatal Depression Scale (EPDS). The scale is comprised of 10 questions that help to identify common symptoms of depression. Each item had four grades that were scored from 0 to 3 according to the severity of symptoms; The total score ranged from 0 to 30. An EPDS score of 13 or above indicated PPD; the scale demonstrated good reliability among Chinese women (validity, 0.79; Cronbach’s α coefficient, 0.87) (Heh, 2001).

### Data analysis

The distributions of associated risk factors were calculated as means for age and as proportions for the other variables. Characteristics were further stratified and tested for differences by ethnic group using the two-sided Student’s *t*-test *for age*, and the *χ*^2^ test for the rest variables. The statistical models assumed that the errors were a binomial distribution, and that significance could be determined by using a likelihood ratio. Multivariable logistic regression analysis of maximum likelihood estimation (forward stepwise) with PPD as a dependent variable, was performed to analyze the main effect of ethnicity after adjusting for potential confounders addressed in this study. To evaluate the associated factors of PPD for different ethnic groups, we estimated the factors’ effects on PPD using multivariable logistic regression stratified by ethnicity. Statistical processing was conducted using SPSS 13.0. A *P*-value of 0.05 or less was considered to be statistically significant.

## Results

### Social-demographic and obstetric characteristics of the women

**Figure 1:**
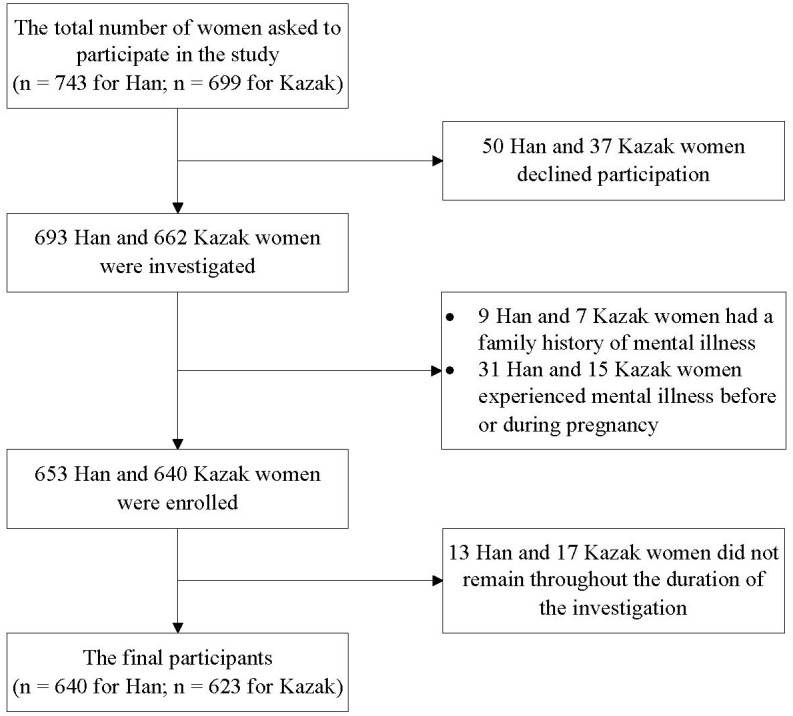
The flowchart of recruitment of women for this study.

A total of 1,442 women were invited to participate. Of those women, 87 (6.0%) declined to participate. The percentage that declined did not vary by ethnic group (50 women (6.7%) for Han and 37 women (5.3%) for Kazak, *P* > 0.05). Of the 1,355 women interviewed, 16 (1.2%) of them had a family history (nine women (1.3%) for Han and seven women (1.1%) for Kazak, *P* = 0.681); 46 (3.4%) of them experienced mental illness before or during pregnancy (31 women (4.5%) for Han and 15 women (2.3%) for Kazak, *P* = 0.025). An additional 30 (2.3%) women were excluded because they did not remain throughout the duration of the investigation. A total of 1,263 women (640 Han and 623 Kazak) were ultimately included in the study. They had a mean age of 28.32 (±5.00) years. The recruitment process is illustrated in Fig. 1.

A significantly higher percentage of the Kazak were religious compared to the Han women (99.0% vs. 8.4%). The percentage of women with a middle school or above education, steady employment, and a monthly household income of more than 2,000 yuan (300 USD)/month was lower among the Kazak (26.0%, 21.7%, and 34.5%, respectively) than the Han (33.9%, 58.1%, and 92.2%, respectively). More Han women were nulliparous (67.2%), preferred cesarean section (48.9%), and received care from their mothers-in-law after childbirth (48.8%), compared to Kazak women (31.9%, 34.2%, and 24.6%, respectively) (Table 1).

**Table 1:** Comparisons of socio-demographic and obstetric variables by ethnicity.

	Overall (*n*= 1,263)	Han women (*n* = 640)	Kazak women (*n* = 623)	*t*/*χ*^2^	*P*
Age (y) (mean ± SD)	28.32 ± 5.00	27.98 ± 4.99	28.68 ± 4.99	2.467^b^	0.014
Religion (yes) *n* (%)	671 (53.1)	54 (8.4)	617 (99.0)	1040.586^c^	<0.001
None/primary education *n* (%)	678 (53.7)	217 (33.9)	461 (74.0)	204.066^c^	<0.001
Stable employment *n* (%)	507 (40.1)	372 (58.1)	135 (21.7)	174.611^c^	<0.001
Household income ≤ 2,000 yuan (300 USD)/month *n* (%)	458 (36.3)	50 (7.8)	408 (65.5)	454.377^c^	<0.001
Smoking history *n* (%)	54 (4.3)	20 (3.1)	34 (5.5)	4.196^c^	0.041
Drinking history *n* (%)	79 (6.3)	60 (9.4)	19 (3.0)	21.540^c^	<0.001
Previous pregnancy loss *n* (%)	314 (24.9)	154 (24.1)	160 (25.7)	0.443^c^	0.506
Multiparous *n* (%)	634 (50.2)	210 (32.8)	424 (68.1)	156.868^c^	<0.001
Unplanned pregnancy *n* (%)	265 (21.0)	144 (22.5)	121 (19.4)	1.804^c^	0.179
Cesarean section *n* (%)	526 (41.6)	313 (48.9)	213 (34.2)	28.135^c^	<0.001
Female infant *n* (%)	576 (45.6)	300 (46.9)	276 (44.3)	0.843^c^	0.359
Mother-in-law as caregiver after childbirth^a^*n* (%)	465 (36.8)	312 (48.8)	153 (24.6)	79.421^c^	<0.001
Breast-feeding *n* (%)	960 (76.0)	444 (69.4)	516 (82.8)	31.319^c^	<0.001
Postpartum UI *n* (%)	315 (24.9)	150 (23.4)	165 (26.5)	1.566^c^	0.211
PPD *n* (%)	184 (14.6)	84 (13.1)	100 (16.1)	2.172^c^	0.141

**Notes.**

a All other caregivers as the reference category.

b *t*.

c *χ*^2^.

UI urinary incontinence PPD postpartum depression

### Multivariable logistic regression analysis for the association of ethnicity and PPD

The overall incidence of PPD was 14.6% (184/1,263) at six weeks after childbirth. PPD was detected more frequently among Kazak women (16.1%) than Han women (13.1%). Kazak women exhibited a higher risk of PPD than Han women (*OR* = 1.561, 95% CI [1.108–2.198], *P* = 0.011) after controlling for previous pregnancy loss, unplanned pregnancy, mother-in-law as a caregiver after childbirth, infant gender, and postpartum UI (Table 2).

**Table 2:** Multivariable logistic regression analysis of the risk factors for PPD.

	*β* coefficient	*P*	*OR*	95% *CI*
Kazak^a^	0.445	0.011	1.561^b^	1.108–2.198
Previous pregnancy loss	0.661	<0.001	1.937	1.362–2.755
Unplanned pregnancy	0.792	<0.001	2.207	1.549–3.146
Mother-in-law as caregiver after childbirth	0.653	<0.001	1.921	1.343–2.746
Female infant	0.605	0.001	1.832	1.302–2.578
Postpartum UI	0.386	0.037	1.471	1.024–2.115

**Notes.**

a Han as the reference category.

b Adjusted for previous pregnancy loss, unplanned pregnancy, caregiver after childbirth, infant gender, and urinary incontinence.

PPD postpartum depression OR odds ratio CI confidence interval UI urinary incontinence

### Multivariable analyses of the associated factors of PPD by ethnic group

Significant risk factors for PPD were previous pregnancy loss, unplanned pregnancy, and female infant. For both ethnic groups they were not different. However, the mother-in-law as a caregiver after childbirth was strongly associated with PPD for the Han women but not for the Kazak women (*OR* = 2.600, 95% CI [1.499–4.512], *P* = 0.001). In contrast, postpartum UI was a significant risk factor for PPD for the Kazak women yet not for the Han women (*OR* = 1.720, 95% CI [1.056–2.804], *P* = 0.003) (Table 3).

**Table 3:** Multivariable logistic regression analyses of the risk factors for PPD stratified by ethnic group.

	Han women				Kazak women			
	*β* coefficient	*P*	*OR*	95% *CI*	*β* coefficient	*P*	*OR*	95% *CI*
Mother-in-law as caregiver after childbirth	0.956	0.001	2.600	1.499–4.512	–	–	–	–
Previous pregnancy loss	0.609	0.021	1.839	1.096–3.087	0.635	0.010	1.888	1.163–3.063
Unplanned pregnancy	0.818	0.001	2.267	1.368–3.756	0.646	0.011	1.907	1.158–3.142
Female infant	0.547	0.043	1.728	1.018–2.933	0.661	0.004	1.936	1.236–3.033
Postpartum UI	–	–	–	–	0.542	0.030	1.720	1.056–2.804
Intercept	−3.160					−2.505		

**Notes.**

PPD postpartum depression OR odds ratio CI confidence interval UI urinary incontinence

## Discussion

The present study represents the first work to investigate and compare the incidence and associated risk factors for PPD among Han and Kazak women in a less-developed region in northwestern China. The incidence of PPD among Han women was consistent with results from an epidemiological survey conducted in Mainland China (Wan et al., 2009) and a literature review of studies conducted in Asian countries that revealed a range from 3.5% to 63.3% (Klainin & Arthur, 2009). PPD among Kazak women in Xinjiang, China has never been documented. Therefore, this work provides an important reference for future research. In summary, this study demonstrated that (1) Kazak women were more likely to develop PPD than Han women, even after controlling for confounders; (2) there was a significant association between postpartum UI and PPD among Kazak but not among Han women; (3) mothers-in-law as caregivers after childbirth was positively associated with PPD among Han but not among Kazak women; and (4) common risk factors for PPD (previous pregnancy loss, unplanned pregnancy, and giving birth to a female infant) existed among both ethnic groups. As this was an exploratory study and there was uncertainty around the sample size estimation, we conducted a post-hoc power analysis. Based on the observed data, with an adjusted *OR* of 1.561 for Kazaks (compared to Hans) and a sample size of 1,263 observations (of which 51% are Hans and 49% are Kazaks), an 82% power is achieved at a 0.05 significance level to detect the association of Kazak ethnicity and PPD.

The current study suggests that UI has a differential impact on PPD risk by ethnic group. Kazak women who experienced postpartum UI had a higher risk of PPD. UI is a worldwide health problem, which adversely impacts quality of life of women (Padmanabhan & Dmochowski, 2014). Pregnancy and birth are known risk factors for the development of UI. The prevalence of postpartum UI is high, affecting as much as 33% of all women (Thom & Rortveit, 2010). Although previous studies have reported a correlation between UI and PPD (Fritel et al., 2016; Hullfish et al., 2007), a better understanding of the differences among ethnic groups with regard to the association of PPD and UI merits further exploration.

The presence of mothers-in-law as caregivers after delivery was found as a risk factor for PPD among the Han, but not among Kazak women. The custom of “doing the month” in China means that females stay at home, do not eat cold foods, and avoid a cool/wet environment in the first month after delivery. It is the family’s responsibility to not only care for the child, but for the new mother as well. Since husbands frequently return to work, the caregiver during “the month” is usually the woman’s mother or mother-in-law. The conflict between mothers-in-law and daughters-in-law is deep-rooted in China. A study by Chi et al. (2016) found that only 14.7% of females felt very satisfied in their relationships with their mothers-in-law. A study in Taiwan, China demonstrated that mothers-in-law produced a negative impact on females during the month (Heh, Coombes & Bartlett, 2004). This study also indicated that Han women who were cared for by mothers-in-law during the month after delivery were 2.54 times more likely to have PPD as compared to those who were cared for by others. The same association did not exist among the Kazak women. We found that both Han and Kazak women share the custom of doing the month. The difference is that almost half of the Han women stay at their home, and are cared for by their mothers-in-law during the month. Because Kazak women traditionally stay at their parents’ home during the month, less than a quarter of the Kazak women in our study were taken care of by their mothers-in-law following childbirth. Therefore, the same negative effects of mother-in-law caregiving would not be observed.

Despite the fact that different associated risk factors were found, risk factors that have been accepted in literature also existed in both ethnic groups, which is previous pregnancy loss, unplanned pregnancy, and giving birth to a female infant. This study confirmed the finding that previous pregnancy loss is an independent risk factor for PPD (Giannandrea et al., 2013). Previous studies also found that in economically underdeveloped countries or regions, unplanned pregnancy represents a risk factor for PPD (De Castro et al., 2015; Lara, Navarrete & Nieto, 2016), as well as women who delivered female infants were more likely to suffer from PPD (Xie et al., 2011). That association between female infant and PPD might best be explained by the prevalent desire for male offspring, and the negative reactions of family members toward the birth of a female infant, although this association was not found in a region with a relatively developed economy in China (Wan et al., 2009).

## Limitations

Several limitations to this study should be noted. First, the screening tool for PPD used in this study was EPDS. Although the Chinese version of this scale has demonstrated good reliability and validity (Heh, 2001), a few Kazak women participants did not understand Chinese and the evaluation was completed with the assistance of Kazak nurses. This may have caused language bias. Second, these findings are not representative of all Han and Kazak women in Xinjiang province, since the purposive convenience sampling method was adopted to recruit equal numbers in each ethnic group. The results could have suffered from selection bias, particularly as those Kazak women who lived in remote areas and did not attend the routine consulting six weeks after childbirth were not included in the study. Finally, the study only included variables regarding socio-demographic and obstetric predictors, interaction of those variables and psychosocial factors could be ignored. Therefore, the results presented here should be interpreted with consideration of these circumstances.

## Implications

The incidence of, and factors associated with, PPD among Han women are well-demonstrated in the literature. However, little is known about the situation in women of ethnic minority in China. Despite the limitations mentioned above, this study has addressed a gap in the literature, and has important implications for future research in related fields. First, given the fact that participants were not randomly selected, replication studies with efforts to minimize the selection bias are encouraged to further validate the results obtained from this study. Moreover, since information about the psycho-social predictors was not collected in this study, the association between ethnicity and PPD requires further examination to provide a comprehensive risk profile. To date, no psychometric instrument has been developed adapted for the Kazak population; studies with qualitative design could be alternative solutions to overcome the lack of validated tools. Beyond that, further research is needed to expand the qualitative aspects of the relationships between mothers-in-law and Han women, or Kazak women’s attitudes about UI. Finally, future research should direct attention to the translation of health-related assessment tools for ethnic minorities in China, thereby providing better medical services for these populations.

## Conclusions

Kazak women were more likely to develop PPD than Han women, even after controlling for confounders. The risk factors for PPD identified were not the same between the different ethnic groups. Future research that explores the relationships between Han women and their mothers-in-law as well as Kazak women’s attitudes toward UI could further understanding about PPD in these populations.

##  Supplemental Information

10.7717/peerj.4335/supp-1 Supplemental Information 1 The raw data of this study

10.7717/peerj.4335/supp-2 Supplemental Information 2 The syntax for the data analyses
