# Supplementary material for: Incidence of and social-demographic and obstetric factors associated with postpartum depression: differences among ethnic Han and Kazak women of Northwestern China
Source: PeerJ. 2018 Jan 29;6:e4335. doi: 10.7717/peerj.4335 (PMC5793707; doi:10.7717/peerj.4335)
Supplement: Supplemental Information 2 [file peerj-06-4335-s002.docx]

**Table 1.**

CROSSTABS

/TABLES=religion education stable_job income＜2000 smoking drinking previous_pregnancy_loss multipara unplanned_pregnancy cesarean_section female_infant caregiver_after_birth infant_feeding postpartum_UI PPD BY ethnicity

/FORMAT=AVALUE TABLES

/STATISTICESAREAN_SECTION=CHISQ

/CELLS=COUNT ROW COLUMN

/COUNT ROUND CELL.

T-TEST GROUPS=ethnicity('0' '1')

/MISSING=ANALYSIS

/VARIABLES=age

/CRITERIA=CI(.95).

**Table 2.**

LOGISTIC REGRESSION VARIABLES PPD

/METHOD=FSTEP(LR) ethnicity age religion education stable_job income＜2000 smoking drinking previous_pregnancy_loss multipara unplanned_pregnancy cesarean_section female_infant caregiver_after_birth infant_feeding postpartum_UI

/CONTRAST (ethnicity)=Indicator

/CONTRAST (religion)=Indicator

/CONTRAST (education)=Indicator

/CONTRAST (stable_job)=Indicator

/CONTRAST (income＜2000)=Indicator

/CONTRAST (smoking)=Indicator

/CONTRAST (drinking)=Indicator

/CONTRAST (previous_pregnancy_loss)=Indicator

/CONTRAST (multipara)=Indicator

/CONTRAST (unplanned_pregnancy)=Indicator

/CONTRAST (cesarean_section)=Indicator

/CONTRAST (female_infant)=Indicator

/CONTRAST (caregiver_after_birth)=Indicator

/CONTRAST (infant_feeding)=Indicator

/CONTRAST (postpartum_UI)=Indicator

/PRINT=SUMMARY CI(95)

/CRITERIA=PIN(0.05) POUT(0.10) ITERATE(20) CUT(0.5).

**Table 3.**

SPLIT FILE SEPARATE BY ethnicity.

LOGISTIC REGRESSION VARIABLES PPD

/METHOD=FSTEP(LR) age religion education stable_job income＜2000 smoking drinking previous_pregnancy_loss multipara unplanned_pregnancy cesarean_section female_infant caregiver_after_birth infant_feeding postpartum_UI

/CONTRAST (religion)=Indicator

/CONTRAST (education)=Indicator

/CONTRAST (stable_job)=Indicator

/CONTRAST (income＜2000)=Indicator

/CONTRAST (smoking)=Indicator

/CONTRAST (drinking)=Indicator

/CONTRAST (previous_pregnancy_loss)=Indicator

/CONTRAST (multipara)=Indicator

/CONTRAST (unplanned_pregnancy)=Indicator

/CONTRAST (cesarean_section)=Indicator

/CONTRAST (female_infant)=Indicator

/CONTRAST (caregiver_after_birth)=Indicator

/CONTRAST (infant_feeding)=Indicator

/CONTRAST (postpartum_UI)=Indicator

/PRINT=SUMMARY CI(95)

/CRITERIA=PIN(0.05) POUT(0.10) ITERATE(20) CUT(0.5).
